# Tribological Properties of 2D Materials and Composites—A Review of Recent Advances

**DOI:** 10.3390/ma14071630

**Published:** 2021-03-26

**Authors:** Bodhi R. Manu, Anju Gupta, Ahalapitiya H. Jayatissa

**Affiliations:** Mechanical Industrial and Manufacturing Engineering (MIME) Department, University of Toledo, Toledo, OH 43606, USA; bodhi.ravindranmanu@rockets.utoledo.edu (B.R.M.); anju.gupta@utoledo.edu (A.G.)

**Keywords:** 2D materials, tribology, thin films, solid lubricants

## Abstract

This paper aims to provide a theoretical and experimental understanding of the importance of novel 2D materials in solid-film lubrication, along with modulating strategies adopted so far to improve their performance for spacecraft and industrial applications. The mechanisms and the underlying physics of 2D materials are reviewed with experimental results. This paper covers some of the widely investigated solid lubricants such as MoS_2_, graphene, and boron compounds, namely h-BN and boric acid. Solid lubricants such as black phosphorus that have gained research prominence are also discussed regarding their application as additives in polymeric materials. The effects of process conditions, film deposition parameters, and dopants concentration on friction and wear rate are discussed with a qualitative and quantitative emphasis that are supported with adequate examples and application areas and summarized in the form of graphs and tables for easy readability. The use of advanced manufacturing methods such as powder metallurgy and sintering to produce solid lubricants of superior tribological performance and the subsequent economic gain from their development as a substitute for liquid lubricant are also evaluated.

## 1. Introduction

Friction is a necessary evil. Friction between the ground and shoes enables walking; however, the friction between the mechanical and electrical components decreases the overall efficiency of their constituting systems [1,2,3,4]. Around 20% of the world energy production is utilized toward overcoming friction [5]. The first documented study of friction was conducted by Thermistius in 350 BC and demonstrated that the rolling friction is less than the sliding friction due to the torque generated by frictional force that assists against friction (µk < µs) [6]. The study of friction requires correlation between theoretical concept, experimental, and numerical investigations, which remain unaddressed. The term tribology was coined in 1960 to connect the three important interdisciplinary fields of research, namely friction, lubrication, and wear. The economic impact of frictional losses was the impetus for eventually connecting these different branches under a common umbrella [7]. This is also critical in expanding the knowledge and filling in the gaps in the literature. Most of the reported literature is experimental in nature, including shared methodologies and original data that further provide novel insights on friction control, the reduction of frictional losses, and a better understanding the mechanisms.

Liquid lubricants are widely used in most industrial applications such as in automobiles and manufacturing industries. Liquid lubricants consist of a base polymer with additives added. The additives provide lubrication, whereas the polymer base provides dispersion and stability to the additives. The additives are very sensitive to the working conditions such as load, speed, and temperature; the viscosity and design of the lubricating system is also important. A good lubricating system design should be able to provide adequate pressure difference for the flow of liquids polymers to the required parts based on viscosity. The narrow range of effective working spectrum and complexity of designing to provide pressure difference for lubricant flow makes it difficult for the use of liquid lubricants in extreme working conditions where temperature of pressure varies substantially or there is the presence of gases and radiations that can react with the lubricant, deteriorating its lubricating property system [8,9].

The solid lubricants were primarily developed for applications where liquid lubricants are inadequate such as in aircraft and space exploration, which is characterized by extreme surrounding conditions in outer space, such as temperature variation during the day, intensity of solar radiations, atmospheric pressure, and atmospheric gasses [10]. Under these conditions, liquid lubricants are inadequate both from the standpoint of stable additive and design constrains. However, very few solid lubricants are used in industrial applications to reduce wear, such as coatings on rollers and balls of antifriction bearings and engine piston assembly. Metal nanocomposite and diamond-like carbon (DLC) coatings are mostly used for these applications.

Graphite is one of the earliest elements used as a solid lubricant or as an additive in liquid lubricants, which further led to an investigation of materials with similar crystal structure as graphite, now known as two-dimensional (2D) materials. These materials are mostly used in solid lubricants and preferred over metals and alloys as they provide easy sliding between the atomic layers and hence provide low friction. MoS_2_, h-BN, and boric acid are some of the successful solid lubricants that fall into this category [11,12,13]. The layered structure of 2D materials is responsible for the materials’ lubricating property in which each layer of these materials is covalently bonded to each other layer through sp^2^ or sp^3^ hybridization, resulting in stronger bonds. This strong interlayer bonding results in 2D materials that are stronger, stiffer, and harder [14,15]. Few 2D materials, such as WS_2_ graphene, possess very low friction coefficients but are susceptible to high wear [16]. In contrast, transition metal oxides, h-BN, and nitrocarbons have superior wear-resistance but a higher friction coefficient [17,18]. This contradictory behavior among the 2D materials is due to the nature of intralayer bonding between each atomic layer. In case of the 2D materials with a very low friction coefficient, the layers are attracted to each other by weak Van der Waals forces, which provides easy sliding between the layers that reduce the friction. On the other hand, 2D metal oxides usually have a hexagonal wurtzite or tetragonal structure in which the interlayer atoms are bonded to each other by covalent bonds, which cause high intralayer friction but reduce wear as it restricts cleavage between layers. With an increase in intralayer bond strength, the coefficient of friction also increases, but the wear on 2D materials decreases.

**Figure 1:**
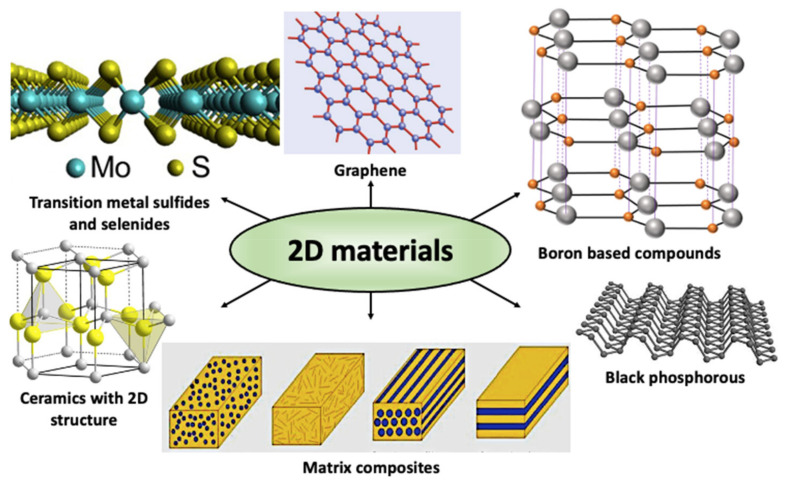
Schematic of different 2D materials covered in this review [19,20,21].

**Figure 2:**
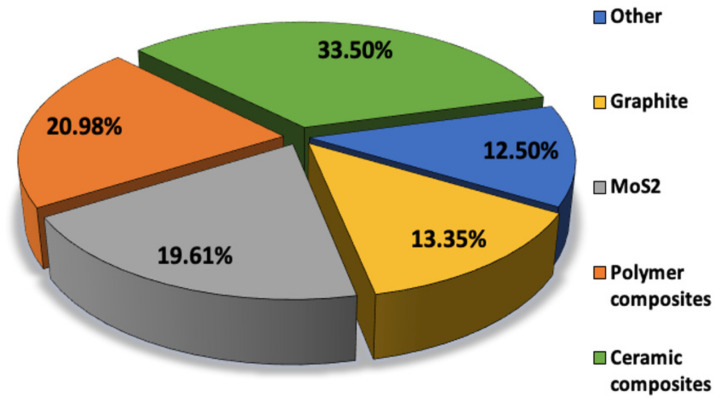
The pie chart shows the research conducted on different solid lubricants from 2000 to 2020; source: Web of Science database, 2000 to 2020. Keywords used in the search: Title = (“solid lubricant” OR “solid-lubricant composite” OR “self-lubricating solid lubricant” OR “polymer, ceramic, and metal matrix composites” and “tribological properties” OR “friction” OR “wear” OR “antifriction”) AND Topic = (“powder metallurgy” OR “compression molding” OR “sintering” OR “UV curing”). The total number of publications analyzed equals 334.

In this comprehensive review, carbon-based solid lubricants such as graphite, graphene, transition metal di-sulfides, and di-selenides such as MoS_2_ and WSe_2_, boron-based compounds -h-BN and boric acid, black phosphorous, and ceramic materials such as ZnO are discussed and summarized in Figure 1 [19,20,21]. A schematic of the relative percentage of publications on these 2D materials is also shown in Figure 2. Among these 2D materials, MoS_2_ and carbon-based compounds including graphite and graphene are the most investigated materials. MoS_2_ is the most successful solid lubricant for aerospace applications in dry conditions, whereas graphite and some other carbon-based lubricants require a humid environment to provide good lubrication [22]. The discovery of graphene and its desirable electronic and tribological properties have led to a detailed investigation on nano- and micro-scale friction for its applications in MEMS/NEMS devices [23,24]. These investigations have provided a deeper insight into friction mechanisms and energy dissipation on an atomic level.

## 2. Mechanisms of Frictional Energy Dissipation on an Atomic Scale

**Figure 3:**
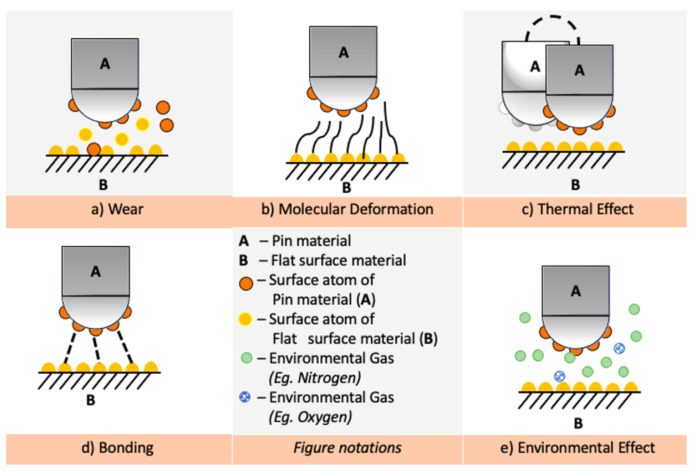
Different microscale mechanisms of frictional energy dissipation: (**a**) Wear mechanism; (**b**) Molecular deformation; (**c**) Thermal Effect; (**d**) Bonding; and (**e**) Environmental effect [28].

Friction at the macroscale is quantified in terms of coefficient of friction and the wear rate. For efficient control of frictional losses, it is important to understand the underlying atomistic mechanisms of frictional losses. Friction results from heat generated due to various physical, mechanical, and chemical interactions occurring at the interface of the sliding surfaces [25,26,27]. However, the complexity of the interactions and the unavailability of a single theoretical model make it impossible to quantify the relative contributions made by each of these interactions. In this section, various known mechanisms underlying heat generation at atomic scale due to sliding are examined to provide a theoretical insight on friction as summarized schematically in Figure 3.

(a) Wear

At macroscale, wear is caused by plastic deformation and fracture of the solid-film materials, which leads to physical damage. Wear makes the surface rougher and further leads to an increase in friction. For solid-film lubrication, it is desirable to have the film undergo ductile deformation rather than brittle fracture. Ductile materials undergo plastic deformation by the movement of dislocations, and it can be controlled by tailoring of grain size and grain orientation. On the other hand, brittle fracture is promoted by crack initiation, and propagation that will lead to a higher wear rate after the onset of the wear phenomenon. The wear debris formed also assists in additional wear especially in the case of brittle films [29,30,31].

**Figure 4:**
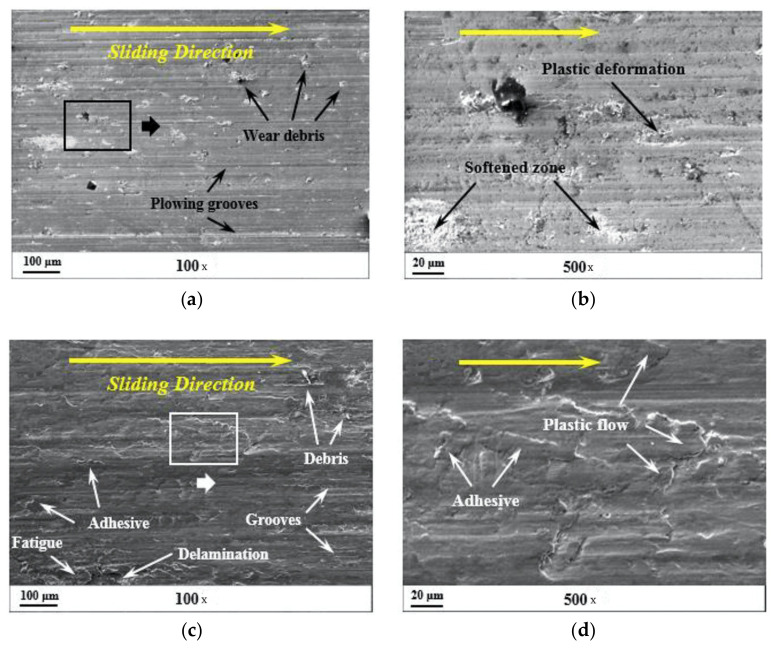
The worn surface morphology of ZrO_2_-toughened Al_2_O_3_ particles reinforced high-chromium cast iron (HCCI) matrix composite at different loads: (**a**) 300 N-low magnification; (**b**) 300 N-high magnification; (**c**) 900 N-low magnification; and (**d**) 300 N-high magnification [32].

Figure 4 shows some of the wear mechanisms found on the worn surface of ZrO_2_-toughened Al_2_O_3_ particles reinforced high-chromium cast iron (HCCI) matrix composite with different forms of wear phenomenon pointed.

(b) Molecular deformation

**Figure 5:**
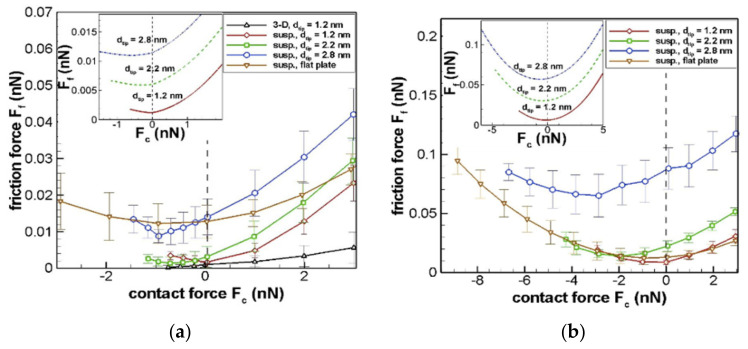
Atomistic simulation result of frictional force( F_f_ ) versus contact force (F_c_) for suspended graphene interlayer adhesion: (**a**) tip sample empirical interlayer adhesion, and (**b**) when tip sample interaction Vander Waal’s force increased by 4 times that of (**a**). Inserts show analytical estimates of F_f_ verses F_c_ different spherical tip diameters. (Here, susp indicates suspended graphene whereas 3-D indicates isotropic graphite [34].

**Figure 6:**
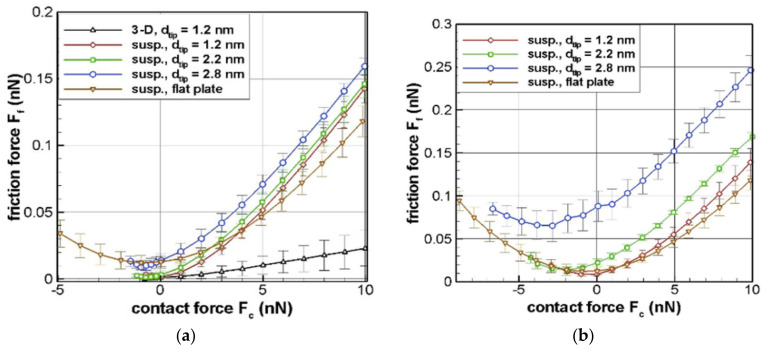
Atomistic simulation result of frictional force versus full range sweep of contact force for suspended graphene interlayer adhesion: (**a**) for empirical interlayer adhesion at a constant Van der Waal’s force and (**b**) for empirical interlayer adhesion when Van der Waal’s forces are increased by 4 times that of (a). (Here, susp. indicates suspended graphene whereas 3-D indicates isotropic graphite [34].

When molecules at the interfaces of the sliding surfaces encounter each other, adhesion of surface atoms of the thin film with the tip of the counter body takes place. 2D materials are known for their low stiffness in the transverse direction [33]. They can easily deform or delaminate depending on the tip-layer adhesion strength. This lateral deformation of 2D layers due to adhesion is known as atomic corrugation. Atomic corrugation leads to molecular deformation and heat generation [34]. Figure 5 and Figure 6 show the molecular dynamics simulation result between friction force F_f_ and contact force F_c_ for suspended graphene and isotropic graphite at nanoscale contact. Simulations were done at different contact tip diameters for Vander Waals adhesion contact/retract and full-range force sweep conditions (experiments), respectively. The analytical values for these simulations are shown as an insert in Figure 5a,b. Figure 5b and Figure 6b show the F_f_ vs. F_c_ relation when tip-sample interfacial Vander Waals forces are increased four times. When the interfacial interaction energy was increased, the magnitude of negative contact load also increased substantially. Negative contact load signifies adhesion between the interfacial atoms due to molecular deformation. Energy is required to break this contact interaction, which is released as frictional heat. The presence of dislocations and inclusion can also increase the corrugation energy as energy is lost as heat to overcome or move these defects. Higher the number of dislocation motion, the higher the heat generated due to molecular deformation [35].

(c) Thermal effect

**Figure 7:**
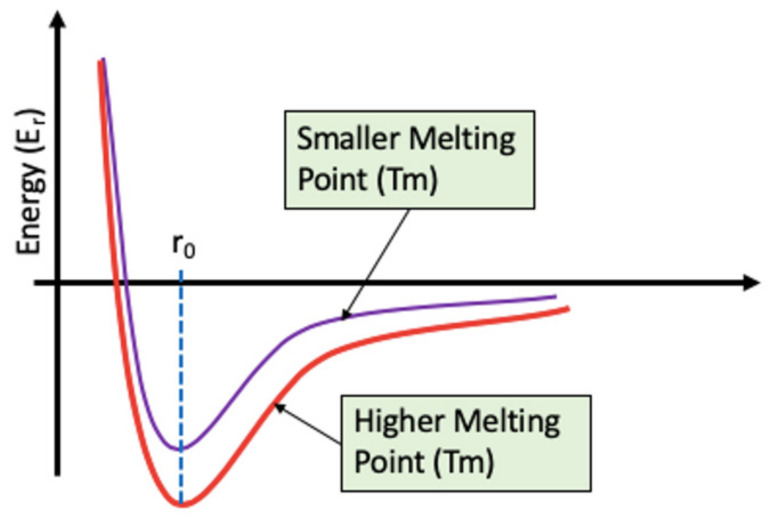
Typical potential energy curve showing the shape of the well with increase in melting point [39].

At a certain temperature, molecules and atoms gain enough energy to move around and across the interface. The rate of these motions can either increase or decrease depending on the surrounding temperature or heat generated due to frictional interaction. The rate of these fluctuation also depends on the interaction potential between the atoms and molecules. When the interaction potential is high, the overall effect of thermal activation on heat generation decreases [36]. In this case, more heat is required to produce these effects. This is attributed to the thermally actuated hooping mechanism of atoms, which ease sliding. The thermal effect of heat generation depends on the sliding velocity and lateral frequency of the sliding interface [37,38]. Figure 7 shows the variation of potential energy with interatomic distance. The higher the depth of the potential well, the more energy is required to overcome the interaction potential, and hence, thermal effect on friction becomes less. In addition, with an increase in depth of the potential well, the melting point of the material increases.

(d) Bonding

One of the most important issues associated with an increase in friction is related to chemical interactions at the asperity contacts. Bonding can occur between the rubbing surfaces or in the presence of environmental reactants. The formation and breakage of these bonds during the relative motion between rubbing surfaces leads to an increase or decrease in friction based on the nature of the bonding energy [40,41].

**Figure 8:**
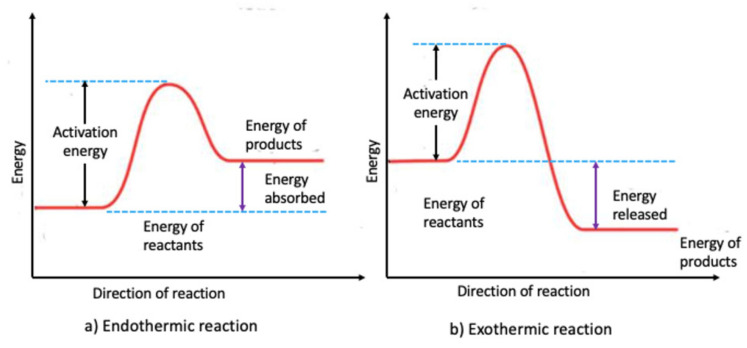
Schematic showing the energy interaction during endothermic and exothermic reactions: (**a**) Endothermic reaction- energy of products is higher than energy of reactants and (**b**) Exothermic reaction- energy of products is less than energy of reactants [42].

The wearing process mostly involves breaking of metallic bonds with high bond dissociation energy and thus produces heat since these reactions are exothermic. In addition, surface oxidation and corrosion as a result of environmental reactants are also exothermic and lead to heat generation. The higher the bond dissociation energy, the higher the heat generated. Figure 8 shows the energy difference between the reactants and products for endothermic and exothermic reaction. For exothermic reaction, the energy of the products is less than that of reactants.

The change in their energy is released into the atmosphere as heat. The binding energy of different types of atomic bonds is shown in Table 1. The higher the binding energy, the more heat is released during bond breakage.

(e)Environment

Solid-film lubrication is highly dependent on the environment. The chemical and physical interactions between the film and the surrounding environment greatly affect the tribological performance [43,44,45]. Table 2 shows the effect of some environmental factors such as humidity and temperature on the lubricating properties of some of the widely used solid lubricants.

## 3. Graphite and Graphene

Graphite is known for its lubricating property from ancient times based on its outstanding qualities as solid lubricant in humid environment. This is due to the weakening of the interlayer Van der Waals forces or the dangling bonds because of the saturation by H+ and OH- ions from humidity [50,51,52]. Therefore, the graphite has been used as an additive in various solid lubricants to improve their tribological performance in a humid environment [53]. Presently most investigation involving graphite as a solid-lubricant focus on improving the wear and friction of different grades of steels for various industrial applications [54,55,56].

Since the discovery of graphene, its electronic properties have gained a lot of attention, and it has been deemed as a promising material that can revolutionize the electronic industry, prompting a substantial number of investigations on understanding its behavior at nano-, micro-, meso-, and macro-scales [57]. Consequently, the electronic, mechanical, and tribological properties of graphene are more established theoretically and experimentally than other novel 2D materials. The tribological behavior of different 2D materials is also affected by their structure, reactivity of different functional groups, chemical affinity to environmental species, and thickness of deposited layers [58,59,60]. Extensive studies on atomistic and molecular scale friction, the effect of external factors, and the influence of addition of atoms or molecules on the electronic and tribological properties of graphene are crucial to an in-depth and systematic understanding of the physics of macroscopic friction [61,62,63]. These studies indicate a clear difference in frictional behavior at macroscale compared with microscale and atomic level. Understanding these microscopic tribological effects is important to achieve super lubricity for practical application over wide ranges of atmospheric and mechanical conditions [64,65].

**Figure 9:**
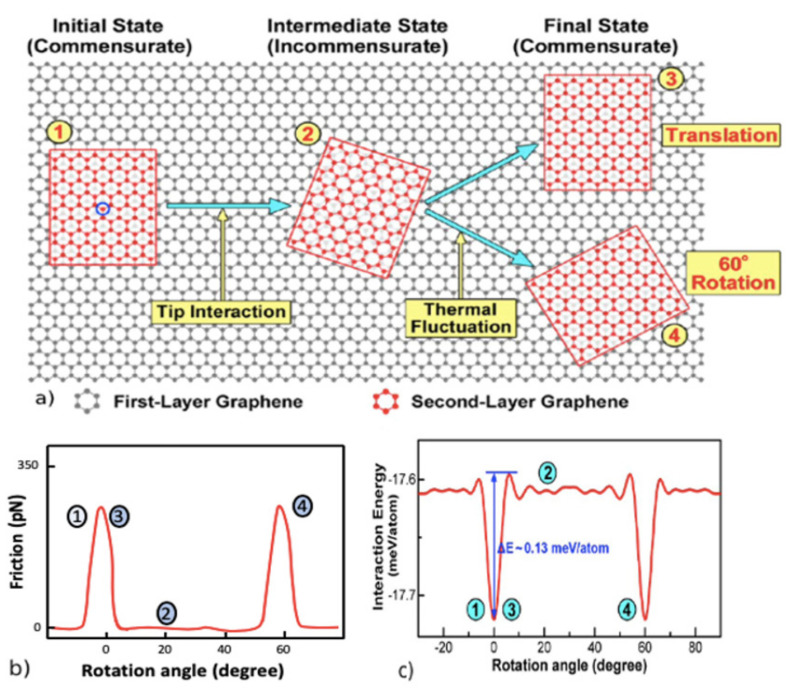
(**a**) Schematic of commensurate and incommensurate sliding of one graphene layer over another [71]; (**b**) variation of nanoscale friction with respect to the change in relative angle of rotation [69]; and (**c**) interaction energy as a function of rotation angle. Negative interaction energy signifies attractive forces and increase in friction increases [71].

**Figure 10:**
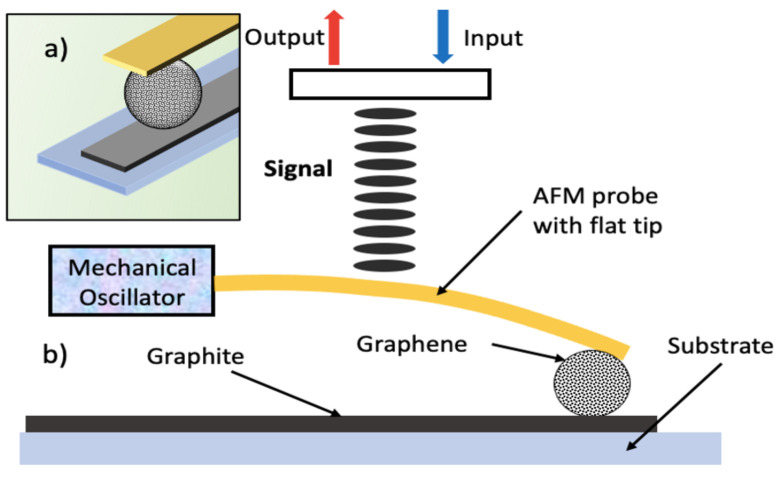
Incommensurate heterostructure interface sliding of multilayer graphene-coated microsphere (GMS) on graphene substrate using tipless mode of atomic force microscope (AFM) (**a**) isometric view and (**b**) schematic of atomic force microscopy of GMS sliding on graphene layers (After [72]).

Investigations on interlayer and intralayer sliding friction are important for understanding the modes of heat generation, dissipation, and their control to achieve super lubricity at macroscale [66,67]. Experimental studies conducted by Kamiya et al., [68] and Dienwiebel et al., [69] on the interlayer sliding of graphene/graphite concluded that the nanoscale friction is highly anisotropic and dependent on the relative angle (rotation angle) of sliding layers as shown in Figure 9. At 0° and 60° angles of rotation, the nanoscale friction and interaction energy indicated by negative sign convention signifying attraction are higher than other angles, and the sliding occurring at these angles is termed as commensurate sliding. If the relative sliding angle is not equal to the commensurate stage, the frictional force is reduced considerably; this phenomenon is known as incommensurate sliding. This incommensurate sliding of graphene and its associated high flexibility in the out-of-plane direction even with thermal excitation [70] has been exploited to achieve macroscopic super lubricity with graphene-coated nanospheres to promote hetero structural interface sliding as shown in Figure 10a.

The friction on heterostructure interfaces was studied by sliding multilayer graphene-coated microsphere (GMS) on different 2D materials using an atomic force microscope (AFM) by Liu et al., [72]. They reported formation of a multiasperity contact with substrates by coated microsphere, resulting in incommensurate interfaces and suggests that superlubric sliding can be achieved for heterostructure multiasperity interfaces. Berman et al., [73] reported to achieve macroscopic super lubricity between graphene and diamond-like carbon due to the formation of nanorolls by a spontaneous wrapping of low stiffness graphene layers during sliding. This produced an incommensurate multiasperity sliding (Figure 10), which was super lubric with a friction coefficient of ~0.004.

Sliding friction on the surface of the graphene is highly dependent on the thickness or the number of atomic layers. With an increase in the number of layers, the friction force decreases and is independent of normal force, sliding velocity, and the tip material. Lee et al., [74] studied the effect of number of graphene layers on friction force using mica substrate, which is highly adhesive to graphene. They reported absence of thickness-dependent frictional effect and attributed it to the decrease in friction with increased graphene layers to the low stiffness of the graphene layers. When graphene interacts with AFM tip in contact mode, graphene adheres to the tip, thus producing a puckering effect and wrinkling, which further increases the friction. The high adhesiveness of the substrate material inhibits the formation of wrinkles, and friction becomes independent of the number of layers, which was later confirmed by various research works that reportedly used atomically thin films subjected to different atmospheric conditions [75,76,77].

Significant research has been conducted on the effect of topology defects [78,79,80,81] and the presence of functionalization groups [82,83] on heat generated due to friction. Atomic steps and step edges results in high friction and heat dissipation due to Schwoebel–Ehrlich barrier [84,85]. Local out-of-plane deformation of the graphene layers increases the number of step edges and lead to additional heat generation [86,87]. The presence of functionalization groups such as hydrogen, fluorine, and oxygen is known to increase the frictional heat, which is assumed to be arisen from the atomic roughening of graphene due to its sp^3^-bonding and increase in atomic corrugation [88] and an increase in out-of-plane stiffness. Ko et al., further showed that hydrogenated, fluorinated, and oxidized graphene exhibited two, six, and seven times increase in friction compared with pristine graphene, respectively [58].

## 4. Transition Metal Sulfides and Di-Selenides

Transition metal sulfides and selenides such as MoS_2_, WS_2_, MoSe_2_, and WSe_2_ are an important class of 2D lubricating materials, especially in space exploration design [89]. These materials are known for their very low friction coefficient in dry and vacuum conditions over a wide temperature range in addition to their stability in radiative environments [90,91,92]. Among these materials, MoS_2_ is extensively investigated and used for astronomical applications. The tribological performance of other transition metal disulfides and di-selenides is comparable to that of MoS_2_ due to their similarity in structure and are also used as a solid-film lubricant [93]. Tungsten-based disulfide and di-selenide are used for high-temperature applications due to their thermal stability and oxidation resistance [94,95]. MoS_2_ is reported to lose its lubricating property around 300 °C due to oxidation, whereas tungsten compounds can maintain lubricity up to 600 °C [96,97]. The lubricious property of these solid films is highly dependent on the microstructure and dopant concentration. The microstructure and their adhesiveness to the substrate are also dependent on the deposition method and deposition parameters such as coating power, time, and the temperature. High-quality films of transition metal disulfides and di-selenides with improved tribological performance produced by using sputter deposition method have also been extensively reported [98]. Various sputtering processes such as co-deposition, multiplayer, and multiphase deposition are used frequently to produce high-quality transition metal lubricious films that can withstand high loads and possess higher wear resistance [99,100,101].

The solid-lubricating properties of these transition materials are known to deteriorate due to the adsorption of atmospheric humidity, the presence of oxygen, and at higher temperatures [43,102,103]. Studies have reported an increase in friction at room temperature due to adsorption of water, which notably does not promote oxidation of MoS_2_. This increase in friction as a result of adsorbed water also restricts the growth of surface tribo-films on the counter surface at room temperature [104]. Curry et al., conducted experiments on highly oriented N_2_-sprayed MoS_2_ and sputtered amorphous MoS_2_ films [105]. The performance of these highly oriented crystal with the basal plane perpendicular to the c-axis showed enhanced performance under humidity and at high temperatures in the absence of molecular oxygen. An increase in temperature and in the presence of oxygen, the surface oxidation of MoS_2_ films leads to an increase in friction coefficient. These oxide films are usually a few nanometers thick and wear off easily, and therefore, exposing the underlying unoxidized MoS_2_ films improves overall tribological performance with time. Thus, it was concluded that adsorption of water molecules reduces the tribological performance of MoS_2_ films compared with the oxidation of a solid film [106,107]. Prasad et al., investigated the quality of the WS_2_ transfer film and noted a deterioration of friction coefficient in the presence 50–60% relative humidity in the air, a trend like that of MoS_2_ [108].

Another characteristic feature of MoS_2_ coatings is the establishment of low friction during the running-in which is enhanced by high loads and can be supported by friction-induced surface crystallization [109]. The formation of a basal-oriented transfer film on the counter-body sliding against a randomly oriented MoS_2_ film has also been reported [110]. Although the reorientation process results in a lower and a stable coefficient of friction, it is shown to significantly decrease the wear resistance of the coating under higher humidity. Tungsten disulfides and selenide show higher thermal stability compared with its molybdenum counterparts. Hence, tungsten-based compounds are known for its lubricating properties at a higher temperature.

Many investigations have been conducted to improve the tribological performance of these films under humidity and for high-temperature applications. Films grown parallel to the substrate (001) are preferred for solid lubrication because their edge sites (step edges), which are more reactive to oxidation and corrosion are masked from the surface. The quality of the synthesized films is also dependent on deposition parameters such as pressure, temperature, and the substrate among other factors. It has been found that sub-stoichiometric films with sulfur and selenide deficiency decreases the probability of re-sputtering, thereby improving the tribological performance [111,112]. An augmented metallic interface between the film and the substrate improves the adhesive property of the film [113,114]. The addition of different materials was also shown to alter the grain structure, orientation, and the performance of the solid film [115]. Doping or co-deposition and using layered films generated through multilayer deposition of various materials are frequently used to improve the resistance of the films under varying humidity and temperature conditions [116].

**Figure 11:**
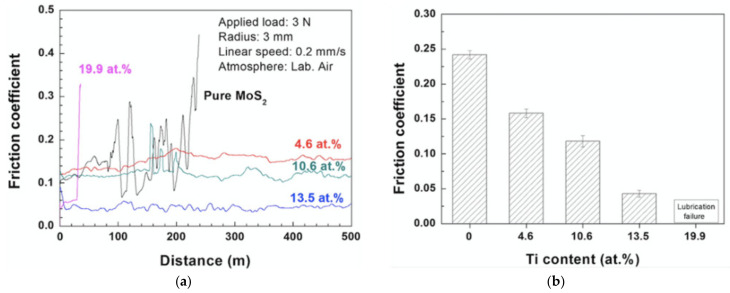
(**a**) Sliding friction curve and (**b**) mean coefficient of friction as a function of Ti content for sputter-deposited Ti/MoS_2_ films [120].

Titanium is a widely used co-dopant in MoS_2_-sputtered films [117]. Rigato et al., [118] reported that the doping of Ti within the layered structure of MoS_2_ increases the distance between MoS_2_ layers and reduces the interlayer friction. Renevier et al., [119] reported that MoS (Ti) produced by close-field unbalanced magnetron sputtering was found to be harder by factors of 1000–2000 than hardness of MoS_2_ (~400 HV) and were much more wear-resistant by a factor of ~100. They were also identified to be lesser sensitive than the atmospheric water vapor, showing an improvement by a factor of 2800 compared with MoS_2_. Quin et al., [120] examined hybrid high-power impulse magnetron sputtered (HIPIMS) MoS (Ti) films and demonstrated that the crystallization degree of the MoS_2_ (Ti) composite coatings decreases with an increase in Ti dopant concentration. This is due to the reactivity of Ti with O that results in the formation of titanium oxides on the surface, inhibiting the oxidation of MoS_2_ and achievement of the lowest coefficient of friction (COF) at 0.04 and at the wear rate of 10 ^−7^ mm^3^ N^−1^ m^−1^ at the optimum Ti content of 13.5% (Figure 11).

**Figure 12:**
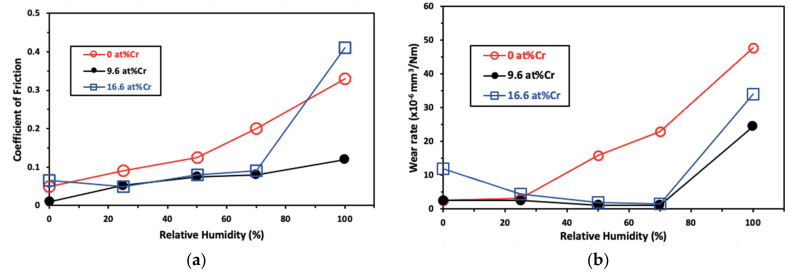
Friction coefficient and wear rate on sputter deposited MoS_2_/Cr composite film as a function of Cr content at different humidity levels: (**a**) variation friction coefficient with relative humidity and (**b**) variation of wear rate with relative humidity [122].

**Figure 13:**
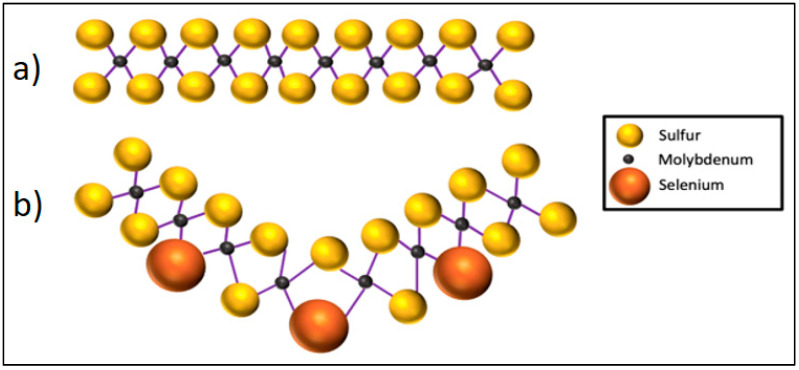
(**a**) MoS_2_ layer and (**b**) substitution of the sulfur atom by selenide leads to a curved structure (after [128]). This increases interlayer distance and reduces friction.

Ding et al., showed that an increase in Cr doping on MoS_2_ led to a substantial decrease in the wear rate and friction with humidity up to 9.6 at% of Cr as indicated in Figure 12. Also, addition of metals such as Au [121], Cr [122], Ni [123,124], Cu [125,126], Zr [127], and metal compounds such as Sb_2_O_3_, WSe_2_ have improved the performance under humid air and vacuum compared with sputter-deposited pure MoS_2_. Doping of WSe_2_ in MoS_2_ films leads to the substitution of sulfur atoms by selenide, which creates a curvature in the linear MoS_2_ structure by increasing the interlayer spacing, as shown schematically in Figure 13 [128]. Few metal dopants facilitate the parallel orientation of basal plane with the substrate in the sliding direction, protect the edge sites from oxidation and further reacts with oxygen to form lubricious metal oxides that improve their performance in humid atmospheric conditions [119,129,130]. The interstitial and substitutional defects increase the interlayer spacing and aids in easy cleavage, thereby reducing interlayer friction.

Doped amorphous carbon and DLC (diamond-like carbon) have also been investigated for improvement in the tribological performance of transition metal dichalcogenides under humid conditions. Notably, the addition of smaller amounts of MoS_2_ in carbon-based compounds facilitate the formation of graphite-like structure [131]. MoS_2_/carbon-doped composite has been highly investigated to improve the strength, wear resistance, and tribological performance of transition metal dichalcogenides in humid environments [131]. Studies have shown that the addition of graphite, r-graphene oxide, DNC, and amorphous carbon to transition metal dichalcogenides improves the tribological performance in humid environments [132].

Multilayer films deposited by sputtering are proven to have superior lubricating properties than the pure solid-film lubricants. The improved performance of these films is due to their increased hardness arising from their distinct superlattice structure of the resultant multilayer films [133]. Mikhailov et al., [134] investigated the performance of multi-films of MoS_2_ using Au, Ni, Pb, or PbO films as the ancillary layer that exhibited higher performance at 50% RH, and it was hypothesized that the improved performance is because the addition of the elements helps in the basal orientation of MoS_2_ and hence improves the performance. In another study conducted by Li et al., [135] involving Pb-Ti/MoS_2_ nanoscale multilayer films produced by sputtering, it was found that the films advanced from a multilayer structure to a composite structure as the bilayer period decreased from 25 to 6 nm due to diffusion of atoms within the bilayer. Hence, there should be a minimum bilayer period thickness to make sure the films formed are multilayer and not composite coating. The nanoscale multilayer film with a bilayer period of 20 nm exhibited superior mechanical and tribological properties than pure MoS_2_, implying that a certain minimum critical bilayer thickness is crucial to produce multilayer films.

**Figure 14:**
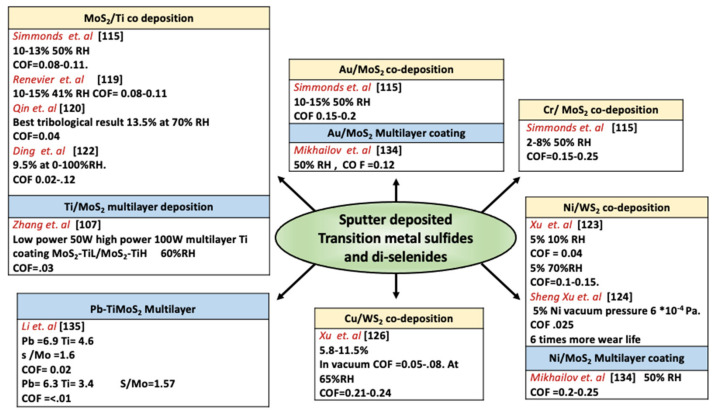
Some publications on co-deposited and multilayer MoS_2_ and WS_2_ films. [115,119,120,122,123,124,126,134,135].

**Figure 15:**
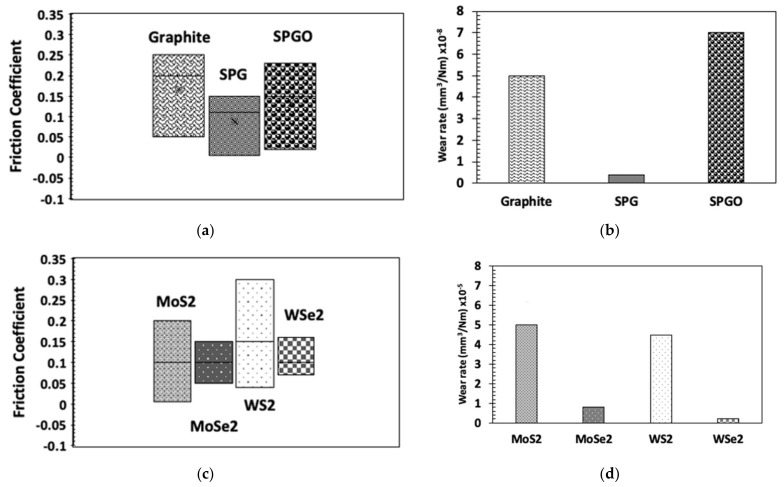
The range of friction coefficient and wear rate for carbon-based films and sputter-deposited transition metal sulfides and di-selenides films: (**a**) Friction coefficient range for carbon-based films; (**b**) Wear rate range for carbon-based film; (**c**) Friction coefficient range for sputter-deposited transition metal sulfides and di-selenides films; and (**d**) Wear rate range for sputter-deposited transition metal sulfides and di-selenides films [102,140,141,142,143,144].

In multilayer deposition, a metallic layer of metals such as Cr and Ti is deposited as the first layer to improve the adhesion of multilayer solid films on to substrate [136]. Hilton et al., formed sputtered Ni–MoS_2_ and Au–(20%) Pd–MoS_2_ multilayer films with appropriate layer spacing and thickness to induce excellent endurance under dry nitrogen and air environments [129]. The thickness of the interlayer distance determines the hardness, microstructure, and superlayer structure, which affects the tribological performance. Many studies report using multilayer and multiphase deposition techniques to find optimum interlayer spacing and composition to identify the best lubricious solid film to address the current needs [99,137,138,139]. Figure 14 summarizes research works conducted on MoS_2_ and WS_2_ co-deposited and multilayer films. The range of friction coefficient and wear rate for carbon-based films and sputter-deposited transition metal sulfides and di-selenides films are shown in Figure 15.

Boron compounds are used extensively in a wide range of tribological applications as an environment-friendly friction modifier and antiwear additive. Both organic and inorganic boron compounds are used as a solid, liquid lubricant [145,146,147] and as a lubricant additive in organic and inorganic compounds [12,148,149]. Boron-based lubricants are mostly explored for industrial application as a solid or ionic liquid lubricant. Among all boron compounds, hexagonal boron nitride and boric acid are investigated as a solid lubricant.

Hexagonal boron nitrides (h-BN) also known as white graphite have been shown to have inferior lubricating abilities compared with graphite and MoS_2_ because of the existence of stronger interlayer Van der Waals forces that restrict the cleavage [150,151,152]. The h-BN has higher temperature stability compared with other solid lubricants and functions well in a humid environment. In contrast to other solid lubricants, h-BN can withstand higher sintering temperature during the powder metallurgical manufacturing processes by virtue of their higher phase-transformation temperature [152,153]. Presently most industrial applications of h-BN focus on improving the tribological performance of different grades of steel for industrial applications [152,154].

Mahathanabodee et al., [152] studied hexagonal boron nitride (h-BN)-embedded 316 L stainless steel (SS316L/h-BN) composites prepared via high-temperature powder metallurgy process and established that increasing h-BN content in the composition leads to a decrease in the hardness, which can be improved by increasing the sintering temperature during the manufacturing process. The best tribological results were obtained for 20vol% of h-BN at a sintering temperature of 1200 °C, which is slightly higher than its melting point of 1250 °C. Avril et al., [155] examined α-Fe (Cr)-h-BN and α-Fe (Cr)-Fe_2_B-FeB films generated on X30Cr13 stainless steel by a laser-melting process that showed an improvement in the tribological properties with the addition of h-BN [156,157]. Miyake et al., [158] investigated the effects of multilayers (C/BN) n films with thickness in nanometric range that were produced by RF sputtering and found the layer thickness of 4 nm showed the lowest friction coefficient of 0.1 at 25 °C.

Zishan et al., [159] explored the temperature stability of SiC and (SiC/h-BN) composite coatings prepared by the pack cementation process on carbon/carbon (C/C) substrate. Their findings supported the addition of h-BN that resulted in stabilization of the friction coefficient of SiC coating at room temperature. At 600 °C, the tribological behaviors of both the coatings showed a higher friction coefficient of 0.75; however, at 800 °C, the SiC coating underwent severe wear and SiC/h-BN composite coating exhibited a lower friction coefficient and wear rate compared with SiC coating. Tyagi et al., [160] also reported a lower friction coefficient for a sintered coating containing h-BN compared with the base matrix and an improvement in lubricating effects at high loads and speeds [161].

**Figure 16:**
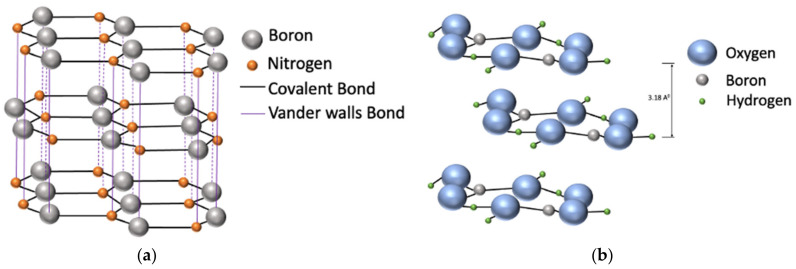
Structure of h-BN and boric acid: (**a**) hexagonal boron nitride and (**b**) boric acid [165].

Boric acid has also been explored as potential solid-lubricating borate due to its graphene like structure as shown in Figure 16. Unlike other solid lubricants, boric acid dehydrates at 170 °C forming oxides that lack the lubricating property. In addition, during sliding at high contact pressure, boric acid solid films can be forced out of the sliding contact area and need to be replenished continuously [162,163]. Due to these issues, boric acid is mostly used in multifunctional systems consisting of solid and ionic liquid lubricants that allows for continuous replenishment of solid-lubricant boric acid crystals [123,164].

## 5. Phosphorus with Layered Structures (Black Phosphorous)

Black phosphorous (BP) is the most thermodynamically stable and nontoxic of all the amorphous forms of phosphorous at room temperature [166]. In recent years, it has gained prominence as a lubricant additive in polymer composites especially with polytetrafluoroethylene (PTFE) as the composite matrix [167]. The addition of BP facilitates the formation of a tribo-film consisting of phosphorus oxide and phosphoric acid over the time that leads to a decrease in friction coefficient and wear as the rubbing progresses [168].

Lv et al., [169] conducted tribological investigations on polyether ether ketone (PEEK)/PTFE and carbon fiber (CF)/PTFE composites with 5% BP nanosheets. The results indicate a dramatic decrease in the coefficient of friction (COFs) and wear rate of both the PEEK/PTFE and CF/PTFE composites in the presence of BP nanosheets. A minimum COF of 0.04 has been reported for both composites with the inclusion of BP. The wear rate of the PTFE/PEEK composite has been observed to decrease with an increase in PEEK concentration, while the wear increased with increasing CF content in CF/PTFE composites. Peng et al., [170] recognized enhanced tribological performance for 0.5 wt.% BP/PTFE compared with 0.5 wt.% BMG (ball-milled graphite)/PTFE. Table 3 enlists the thermal stability and temperature range of the applications of some solid lubricants.

## 6. Ceramics in Tribology Research

Ceramic materials with tetragonal and wurtzite structures have been used recently as a solid lubricant for high-temperature applications. Unlike traditional solid lubricants with 2D structure, these materials have stronger intralayer and interlayer covalent bonds. The intralayer covalent bonds is responsible for the brittleness and hardness of the ceramics, and as a result of their brittle nature, the lubricating property of ceramic materials is known to depend heavily on their microstructure under varying temperatures [172,173,174,175]. In the case of ceramic materials, the wear and failure happen due to crack initiation and propagation. Karch et al., [176] successfully demonstrated the achievement of ductile fracture of TiO_2_ ceramics at low temperature for TiO_2_ ceramic by controlling the stoichiometry, grain size, and crystal orientation both experimentally and analytically. With the advancement in thin-film coating technologies that offer precise control of these deposition process parameters, ceramics can emerge an alternative for high-temperature and corrosive environmental applications.

It has also been shown that the sub-stoichiometric oxide coatings with oxygen vacancies can lead to the development of new crystallographic shear systems due to diffusion creep; consequently, the shear strength and friction coefficient can be controlled by controlling the stoichiometry [177]. The grain size of the oxide films further aids in reducing friction. For instance, the reduced friction of the nanocrystalline ZnO films is attributed to their sub-micrometer-scale spherical nature that enables a shift in their contact configuration from sliding to rolling. However, these sub-micrometer-sized films possess poor antiwear properties due to their low hardness, which can be improved by inclusion of other elements in films such as nitrogen and carbon. These particles have been shown to squeeze into the grooves on the rubbing surfaces to reduce wear. Nitrogen in particular has been extensively used for reducing the grain size of ZnO. For most oxides, epitaxial growth along the (0001) plane generates the lowest friction coefficient as it has the lowest energy of all the possible crystallographic orientations, which is also known to reduce friction [178,179]. The addition of noble metals such as silver and gold also causes these metals to precipitate along the grain boundary thus strengthening the grain boundaries. This reduces diffusion and dislocation along the grain boundary, improves the hardness, and reduces friction [180,181].

## 7. Metals, Ceramic, and Polymer-Based Matrix Composites

Recently, there has been a significant number of investigations using new and advanced manufacturing methods such as powder metallurgy and 3D printing, centered on understanding and improving the tribological performance of solid lubricants. Improving the wear life of solid lubricants has been one of the key challenges in the field of tribology for their reliable industrial and engineering applications. These advanced manufacturing processes help in controlling the microstructure and lubricant concentration with greater precision. Another recent trend in solid-lubrication research is the focus on the manufacturing and characterization of composites comprising of solid lubricants to reduce friction that has been dispersed in a hard matrix to lengthen the wear life [137].

**Figure 17:**
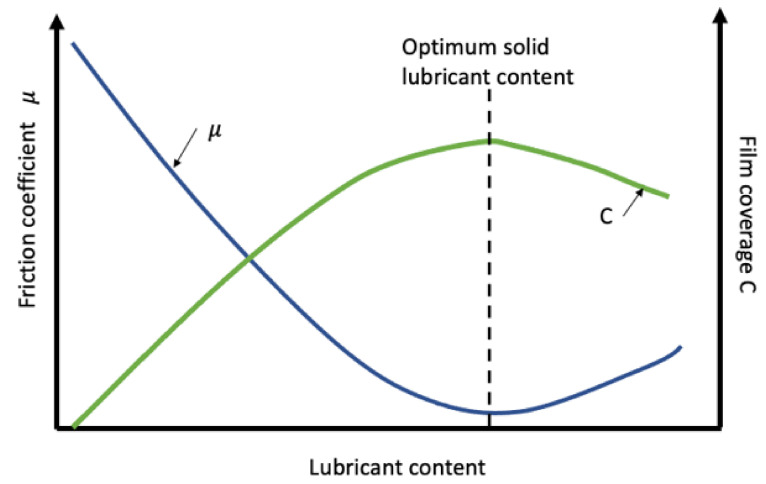
Relationship between film coverage and friction coefficient as a function of solid-lubricant content for MoS_2_ films [21].

As discussed previously, metal, ceramic, and polymer matrix composites are widely explored as solid lubricants. Currently adopted matrix materials typically consist of metals such as copper [182], steel, and aluminum [183]; ceramics like aluminum oxide and silicon carbide, and polymers including PTFE, polyamides [184,185]. Solid lubricants such as MoS_2_, carbon black, graphite in addition to nanometric scale carbon fibers, titanium, and many more have been investigated with varying composition and a variety of manufacturing parameters, namely sintering temperature and powder size to determine solid-lubricant films with advanced tribological properties and reliability for industrial applications [21,169,186]. Figure 17 correlated the film coverage and friction coefficient as a function of solid-lubricant content for MoS_2_ films. A substantial number of publications has reported a variety of combination of different solid lubricants and their matrices to generate the best possible solid-lubricant films. Table 4 summarizes some pioneering work that used matrix composites for solid lubrication in the past two decades.

Lightweight components are the biggest needs of automobile and aircraft industries that remain a challenge to this date. Aluminum and titanium have been explored as an alternative to heavy-duty steel to produce machine parts [187,188]. Reinforcement of metals and alloys along with solid lubricants, hard ceramic particles, and fibers have been an area of interest in the development of MMC with precise balances of mechanical, physical and tribological characteristics [189,190]. Metal matrix composites reinforced with carbon, polymer, or ceramic fibers with varying compositions of solid-lubricant content have been investigated extensively for spacecraft applications [21]. MoS_2_, as the most successful solid lubricant in space applications, has gained a lot of eminence in the field of solid-lubrication research. Additionally, some investigations reported the use of Ni, WC, c-BN, and graphite solid-lubrication coatings on softer materials such as aluminum alloys to increase the surface hardness [190,191,192]. By contrast, the focus of automobile industries has primarily been on mixed lubrication, which is the combination of solid and liquid lubricants to address their tribological focused challenges.

A considerable amount of work has been conducted on copper, iron, nickel, and aluminum metal matrix composites with MoS_2_ as the primary lubricating material [193]. The high thermal and electrical conductivity of copper has led to its applications in electrical contacts, bearings, slide blocks, bushes, and other friction materials. Copper is known to be reactive toward MoS_2_ that results in the formation of Cu- Mo mixed sulfides that deteriorate the strength and tribological properties [194]. Addition of alloying elements in Cu to form brass and bronze helps in overcoming the reactivity of MoS_2_ with Cu [195,196,197]. Iron and steel have been dominating the manufacturing and automobile industries since the industrial revolution (~1800 AD), particularly steels due to their high resistance against corrosion, fatigue, and wear. The reliability and availability of different grades of steel powders [198,199] have led to advancement of sintered steels that are further alloyed with elements and ceramics to control the chemical composition and the processing parameters, to achieve iron-based MMC with enhanced compressibility, machinability, hardness, strength, and toughness [200,201]. Nickel is used in matrix for high-temperature applications such as turbine engines, radiator systems, and nuclear reactors [202,203]. The use of solid lubricants for high-temperature applications is challenging, and therefore, MMC containing Ni matrix comprising of two or more solid lubricants such as graphite, Ti_3_SiC_2_ WS_2_, and PbO [204,205] is used for lubrication at a wide range of temperature applications [206]. In recent times, aluminum and titanium have gained attention as a potential solid-lubricating matrix due to their light weight and specific strength [207,208]. Some investigations have reported the use of a silver matrix for the lubrication of electrical contacts [209,210].

Ceramic materials are known for their high-temperature stability and chemical inertness. Solid-lubricant powders possessing high thermal stability such as BaF_2_, CaF_2_, SrSO_4_, and CaSiO_3_ are mixed with ceramic powders such as ZrO_2_, Y_2_O_3_, Al_2_O_3_, and TiC, and sintered at high temperature and pressure to produce lubricious ceramic composites [211,212,213]. Ouyanga et al., found that unlubricated ZrO_2_ (Y_2_O_3_)–Al_2_O_3_ has a friction coefficient of 1.15 and a wear rate in the order of 10−4 mm^3^ N^−1^ m^−1^ at 800 °C [211]. The addition of SrSO_4_ reduces the brittleness of the ceramic matrix and facilitates plastic deformation. It is also reported that the effective spreading of lubricating material over the ceramic matrix is responsible for reducing the friction and wear over a wide range of temperatures. The size of the ceramic powder and sintering temperatures also affects the tribological performance [211,214,215].

Solid lubrication using polymeric materials such as polyamides, PMMA (Polymethyl methacrylate), and PTFE (Polytetrafluoroethylene) have also been used as a lubricating matrix in automobile body parts. The manufacturing feasibility arising with the addition of nanofibers enhances strength, and the presence of solid lubricants reduces friction, which has further prompt investigations on the applications polymer matrix composites [216]. The discovery of polymer nanocomposites and graphene has led to an interest in developing polymer/graphene nanocomposites with superior mechanical, thermal, gas barrier, electrical, and tribological properties [217,218]. A significant number of reports are centered on various polymer nanocomposites and the impact of fiber reinforcement and lubricant that influences on the tribological performance [219,220,221,222,223].

## 8. Conclusions

The importance and current research on solid-film lubrication have been extensively reviewed to provide a holistic understanding of its importance, advantages, and limitations regarding its application in the industry. The effect of various alloying elements, manufacturing processes, and associated process parameters on the tribological performance of solid lubricants, such as carbon-based and molybdenum-based compounds, is discussed from both a theoretical and experimental investigation standpoint. Molybdenum-based compounds are used extensively in astronomical and aeronautical applications; however, the lubricating property of MoS_2_ is susceptible to atmospheric humidity. On the contrary, carbon-based solid lubricants are known for their advanced tribological performance under humid conditions due to the weakening of interlayer Van der Waals bonds due to saturation of H^+^ and OH^−^ ions from water, which facilitates their interlayer cleavage. A comprehensive review on 2D materials like h-BN, boric acid, and black phosphorous is also covered from an application perspective due to their recent prominence in the literature. Based on the high thermal stability of h-BN, it is deemed the preferred primary lubricant to produce lubricious matrix composites at higher sintering temperatures. Boric acid requires replenishment at intervals, and therefore, used multifunctional solid and ionic liquid lubricants involved in the dispersion of boric acid in liquid lubricants. Black phosphorous is used in lubricious polymeric matrix composites for bearing cages, gears, and cams in the automobile industry. The exploitation of powder metallurgy and sintering methods to produce ceramic and metal matrix composites for high-temperature, corrosion-resistant, and high-wear applications is also discussed. Notable case studies on sputter-deposited and sintered solid-lubricant composites, with reported values of coefficient of friction and environmental conditions, are summarized in tables and graphs for easy readability and understanding.

Solid lubricants possess different desirable properties in comparison with liquids. The higher working range and lubricating properties for extreme conditions are the biggest advantages in commercial applications. However, the lower wear life and difficulty in recoating are the biggest challenges for being considered as a replacement of liquid lubricants. Some solid lubricants such as diamond-like carbon coatings are used along with liquid lubricants to provide better lubricant performance. The present market for solid lubricants is still on extreme condition lubrication, but solid-lubricant nanocomposite coatings have gained some momentum in high-performance industrial applications, such as in mining and wind turbines. Solid-lubricant coatings can be used for many applications when liquid lubricants alone cannot provide the required lubrication need. The increased demand for green or environment-friendly energy systems such as electric engines and wind energy harvesters will lead to the increased use of solid lubricants, as some additives in oil lubricants are known to be environmentally hazardous.

## Figures and Tables

**Table 1:** Binding energy and melting temperature of different bond types [39].

Bonding Type	Substance	Binding Energy (Kcal/mol)	Melting Temperature (°C)
Ionic	NaCl	153	801
	MgO	239	1000
	Si	108	1410
Covalent	C	170	>3550
	Hg	16	−39
	Al	77	660
Metallic	Fe	97	1538
	W	203	3410
Van der Waals	Ar	1.8	−189
	Cl2	7.4	−101

**Table 2:** Effect of environmental factors on the lubricating properties of some known solid lubricants.

Humidity	MoS_2_ and diamond like carbon (DLC) friction increases with humidity Graphene, h-BN, and ultra-nanocrystalline diamond (UNCD) friction decrease with humidity due to saturation of dangling bonds [22] In dry condition (0% relative humidity, RH) hydrogenated DLC shows super lubricity (COF < 0.01) due to shear-induced structural changes [46]
Nitrogen	h-BN loses lubricating property hydrogenated DLC show super lubricity with trace water content (120 ppm) [47]
Oxygen	h-BN loses lubricating property due to oxidation MoS_2_ is also susceptible to atomic oxygen and decreases the lubricating effect
Hydrogen	Improves the tribological performance of amorphous nonhydrogenated DLC by hydrogen termination For hydrogenated DLC, the presence of small amount of water (120 ppm) increases friction [48]
Temperature	For MoS_2_ COF decreases with the increase in temperature due to desorption of water up to 300 °C. At 400 °C, oxidation of MoS_2_ causes deterioration of tribological properties WS_2_ and WSe_2_ can resist oxidation up to 700 °C
Vacuum	h-BN loses lubricating property DLC and diamond shows high friction due to desorption of hydrogen [49] water desorption deteriorates the lubricating property of graphite and graphene

**Table 3:** Thermal stability and temperature range of application of some solid lubricants’ [165,168,171].

Material	Temperature Range (°C)	Temperature of Thermal Stability (°C)	Friction Coefficient
MoS_2_	−184–400	350	0.05–0.25
WS_2_	−184–454	425	0.05–0.25
Graphite	−184–650	500	0.1–0.3
h-BN Boric Acid Black Phosphorous	−184–538 20–80	700 170	0.1–0.2 0.05–0.07 0.2–0.33

**Table 4:** Some publications on using matrix composites for solid lubrication from 2000 to 2020.

Publication	Matrix	Lubricant	Parameters	Important Findings
**Chen et al., 2008** [150]	Copper	Graphite from 10 wt.% to 0 wt.% h-BN from 0 wt.% to 10 wt.%	Block-on-ring wear tester Counter surface: AISI52100 bearing steel	The lubrication effects of graphite are superior to those of h-BN. Added graphite with low contents of h-BN can stabilize friction and wear properties COF increased from 0.35 to 0.55 with an increase in load from 50 to 125 N
**Tang et al., 2011** [224]	Copper	NbSe_2_ nanofiber	Ball-on-disk, 50–70% RH Counter surface: 440-C stainless steel	15 wt.% NbSe_2_ nanofibers showed a very low coefficient of friction of 0.1487
**Zhang et al., 2019** [225]	Copper	Ni/NbSe_2_	Ball-on-disk Counter surface: GCr15	15 wt.% Ni/NbSe_2_ showed the lowest friction coefficient (0.16) and wear rate (4.1 × 10 ^−5^ mm^3^ N^−1^ m^−1^)
**Sundararajan et al.** **2016** [226]	Cu-X SiC wt.% (X = 0, 5, 10, and 15 wt.%)	Gr at 5 and 10 wt.%	Pin-on-disc	10 wt.% gr and 15 wt.% SiC best result
**Prabhu et al., 2015** [227]	Cu-20 vol.% silica	10 vol.% of MoS_2_ or graphite or h-BN	Disc-on-pad dynamometer, 70% RH Counter surface: gray cast iron disc	MoS_2_ is the most effective lubricant with COF = 0.18−0.3 for sliding speed for 3–9 m/s followed by, graphite, and h-BN
**Zhen et al.** **2017** [228]	Nickel	Ag = 12.5 wt.% Baf_2_/CaF_2_ = 5 wt.%, Graphite = 0, 0.5, 1, 2 wt.%	Ball-on-disk, RT to 800 °C Counter surface: Si3N4 ceramic ball	0.5 wt.% graphite exhibited the lowest COF at different testing temperatures except at 800 °C. The composite with 2.0 wt.% graphite showed the lowest at 800 °C
**Zhao et al.** **2019** [229]	Nickel	h-BN=1.25 wt.% nano-Carbon = 5 wt.%	Experiments conducted from 25 °C -500 °C	The encapsulation of h-BN with nano-Cu increased the h-BN content in the coating COF reduced from 0.48 to 0.35 from 25 °C to 500 °C
**Chen et al.** **2018** [230]	Ni Cr 80–20 wt.%	MoS_2_ Graphite	Pin-on-disk Counter surface: steel disk (Cr12MoV)	10% MoS_2_ + Ni + Cr (80–20 wt.%) showed best result with COF=0.02
**Huang et al., 2017** [231]	WC-Ni-Cr88 wt.% WC, 11 wt.% Ni and 1 wt.% Cr	WS_2_= 5 wt.%	Ball-on-disk Counter surfaces: WC-Ni balls	Composite sintered at 950 °C under 250 MPa showed best COF = 0.13
**Kulka et al.** **2019** [232]	Ni	CaF_2_ 20%	Pin-on-disc Counter surface: Inconel 625-alloy	COF =0.75 to 0.45 from room to 600 °C
**Gupta et al., 2019** [233]	Fe-0.3C-2Ni based composites	WS_2_ (3, 5, 7 and 9 wt.%)		The composite with the highest amount of WS_2_ (9 wt.%) showed the lowest coefficient of friction (0.47)
**Zalaznik et al., 2016** [234]	PEEK	micro and nano MoS_2_, WS_2_	Pin-on-disc Counter surface: stainless steel pin (100Cr6).	Friction reduced from 0.6 to 0.4 with the addition of micro and nano MoS_2_, WS_2_
**Cura et al., 2013** [235]	Al_2_O_3_ – 15 wt.% ZrO_2_ (AZ)	3 wt.% of CaF_2_, BaF_2_, MoS_2_, WS_2_, h-BN, or graphite	Scratch testing at 27N	AZ COF=0.068 CaF_2_ COF=0.043 BaF_2_ COF=0.082 MoS_2_ COF=0.086 WS_2_ COF=0.093 h-BN COF=0.086 Graphite COF=0.121
**Ali et al.** **2019** [236]	M50 steel	TiO_2_ 10 wt.% TiO_2_/G powder (10 wt.% TiO_2_+5 wt.% G)	Pin-on-disk Counter surfaces: Si3N4 balls	The tribological performance of TiO_2_/G was the best It decreased from 0.43 to 0.2 from 25–450 °C Pure M50 had a COF of 0.8
**Tao et al.** **2001** [237]	Tin-bronze	Graphite, MoS_2_ or PTFE	Contact pressure = 5 MPa Sliding velocity = 0.16 m/s Testing time = 8 h	PTFE 20 wt.% and graphite 40 wt.%. showed the best result with COF=0.13 Wear rate also reduced by three orders of magnitude for the above sample
**Mushtaq et al.2018** [238]	Fe–Cu 5 wt.%–Sn3 wt.%	MoS_2_ (0-3 wt.%)	Ball-on-disc Counter surface EN-8 steel	Increasing MoS_2_ content from 0 to 3 wt.% the coefficient of friction decreased from 0.85 to 0.25.

## Data Availability

Data sharing is not applicable for this article.
